# Reef Fish Survey Techniques: Assessing the Potential for Standardizing Methodologies

**DOI:** 10.1371/journal.pone.0153066

**Published:** 2016-04-25

**Authors:** Zachary R. Caldwell, Brian J. Zgliczynski, Gareth J. Williams, Stuart A. Sandin

**Affiliations:** 1 Scripps Institution of Oceanography, Center for Marine Biodiversity and Conservation, La Jolla, California, United States of America; 2 The Nature Conservancy, Hawaii, Honolulu, Hawaii, United States of America; 3 School of Ocean Sciences, Bangor University, Menai Bridge Anglesey, United Kingdom; Department of Agriculture and Water Resources, AUSTRALIA

## Abstract

Dramatic changes in populations of fishes living on coral reefs have been documented globally and, in response, the research community has initiated efforts to assess and monitor reef fish assemblages. A variety of visual census techniques are employed, however results are often incomparable due to differential methodological performance. Although comparability of data may promote improved assessment of fish populations, and thus management of often critically important nearshore fisheries, to date no standardized and agreed-upon survey method has emerged. This study describes the use of methods across the research community and identifies potential drivers of method selection. An online survey was distributed to researchers from academic, governmental, and non-governmental organizations internationally. Although many methods were identified, 89% of survey-based projects employed one of three methods–belt transect, stationary point count, and some variation of the timed swim method. The selection of survey method was independent of the research design (i.e., assessment goal) and region of study, but was related to the researcher’s home institution. While some researchers expressed willingness to modify their current survey protocols to more standardized protocols (76%), their willingness decreased when methodologies were tied to long-term datasets spanning five or more years. Willingness to modify current methodologies was also less common among academic researchers than resource managers. By understanding both the current application of methods and the reported motivations for method selection, we hope to focus discussions towards increasing the comparability of quantitative reef fish survey data.

## Introduction

Reef fish populations are changing on a global scale as a result of habitat degradation, pollution, and fishing [1–6]. In order to understand the status and trends in reef fish populations, quantitative techniques are employed to assess and monitor key characteristics of fish assemblages [7–10]. In many of the world’s reefs, assessment relies solely on catch-based sources of data, including both fishery-dependent and fishery-independent approaches [11]. Such sources of data are valuable as they provide a snapshot of the fish taxa that are harvested using particular gear types. However, catch-based data have a limitation in that they have gear-specific sampling bias, most prominently affecting the range of taxa, trophic levels, and size classes that are sampled [12]. For example, while hook-and-line fisheries tend to land larger-bodied predatory species on coral reefs, traps and nets are much less selective with landings encompassing broader size classes and trophic levels [7,8,10]. In nearshore habitats, fishery-independent, diver-survey-based data can provide a valuable and cost-effective tool to assess a wide range of fish taxa and size classes [13].

Fishery-independent, *in situ* survey-based methods, including underwater visual censuses, are widely used to estimate abundance and biomass of fishes. In sampling both target and non-target fisheries taxa, these methods have increased our understanding of the ecological effects of fisheries exploitation and environmental disturbance of nearshore ecosystems [14–20]. The goal of *in situ* surveys is relatively consistent regardless of method used, namely to characterize and quantify the composition of the fish assemblage (i.e., abundance, size structure, and species composition). Survey data can also be used to estimate the number of individuals of each species of interest within a defined area, making it possible to derive estimates of density (numbers of individuals per unit area) and diversity for the sampled taxonomic group [13,21]. Furthermore, for a growing number of applications, surveyors also collect data on the size of individuals in the survey area, enabling the study of size distributions and estimation of biomass density (calculated from density estimates and species-specific length-weight relationships) [14,17,22]. Size-specific data with interpretations in units of biomass are extremely important for the study of fishes, given the indeterminate nature of fish growth and size-specific variation of fish functioning (e.g., fecundity, predation threat) [23].

Despite the many benefits of *in situ* fish surveys, certain limitations and operational challenges merit attention. For fishes, a suite of behavioral and morphological characteristics influence the species-specific efficacy of sampling approaches [24,25]. In most cases, diurnally active species are the primary focus, given that most surveys are conducted in daylight hours when many nocturnally active species are sheltered and thus may be difficult to enumerate accurately. Further, a suite of diurnally active species and size classes use crypsis to avoid detection by predators, including strategies of camouflage and of hiding within the reef substrate. For many non-lethal survey protocols, including visual census, cryptic species are underreported relative to taxa that are larger or more mobile [26]. Finally, the level of activity of individuals can influence the likelihood of detection. Whereas for sedentary species detection is based upon the observer finding the individuals, for more mobile species the speed of movement can influence detectability with potential of generating overrepresentation of very fast-moving species by passing within detection windows multiple times [24]. Detection of more mobile species can also be impacted by the fish’s response to the divers’ presence. Some species, especially those that are targeted by fishers, have been observed to avoid divers [25,27–30], while other species have the tendency to be attracted to diver presence and noise [25,31,32]. These behavioral traits of fish species can alter the number of individuals recorded by divers and provide inaccurate information on species abundance [25,27,33].

Most survey methods have been constructed to minimize bias and maximize accuracy and precision of enumerations for a target group of taxa, all within the operational constraints of time and cost efficiency [24,25]. Across most diver-based methods, the principal axes that are varied are the survey area and the movement patterns of the observer. Table 1 provides an overview of the most commonly employed methods and offers a summary of the reported strengths of each. Operationally, however, most methods used for community-wide assessments of reef fish assemblages that were recorded in this study consist of some form of belt transect, stationary point count, or timed swim, with there being variations of each [25]. Definitions of these three methods can be found in S3 File.

**Table 1:** List of underwater visual census (UVC) methods commonly used to quantify reef fish assemblages, their target taxa, associated sampling area, biases associated with movement pattern of the observer, and their principal reported strengths and weaknesses as evaluated in the literature.

Method	Target taxa	Area sampled	Movement of diver	Reported Strengths	Reported Weaknesses	Key References
Belt transect (strip/line transect)	Assemblage-wide	50–300 m^2^	Low to medium	Captures high diversity, easy to execute, low border effects, repeatable, not greatly affected by habitat variation, visibility variation, or variability between divers, captures species with patchy distributions, widely used in past studies, effective in low visibility and highly rugose habitats	Can underestimate cryptic fishes if swath is too wide, can underestimate presence of cryptic species, larger species may leave survey area, training can be time consuming,	[13,24,44,58–65]
Stationary point count	Assemblage-wide	50–300 m^2^	None to low	High efficiency, eliminates diver movement/search biases (if instantaneous), captures mid-water species, effective at capturing nearshore pelagics	Can underestimate cryptic fishes if radius is too wide, training can be time consuming	[21,24,33,59,62,64,66,67]
Timed swim (Roving diver)	Assemblage-wide	50–1000 m^2^	Medium to fast	Captures high diversity, highly portable, low equipment requirements, quick to employ	Cryptic species are often underrepresented, sometimes difficult to measure entire assemblages, cryptic species are often underrepresented, sometimes difficult to measure entire assemblages, challenges in density estimate if area surveyed is not estimated accurately	[58,68–70]
Towed diver	Large-bodied fishes (generally >50 cm TL)	1000–25,000 m^2^	Fast	Captures highly mobile species, island-scale assessment, works well under wave-exposed conditions	Cryptic species underestimated, difficult to measure entire assemblages, tendency to attract larger predatory fishes	[71–73, 82]
Video (remote, baited, laser videogrammetry, stationary, stereo, and towed)	Community wide and large-bodied predatory species	1–10 m^2^	NA	Deeper depths can be surveyed, captures large-bodied individuals, captures mobile predators, works in low visibility conditions, removes diver effects, robust to denuded assemblages, cost-effective, provides permanent record	Baited cameras tend to attract piscivores and carnivores, diver operated cameras can make sizing smaller species difficult, traditional diver-based methods seem to collect greater species richness, inconsistent census area	[33,64,74–81]
Distance Sampling	Assemblage Wide	1–10 m^2^	NA	Gives accurate representation of fish abundance, all fishes within sight along a transect or point will be sampled and recorded	Distances may be difficult to determine	[36,64]

Although each method of visual assessment is based on a similar premise of unbiased enumeration, there are real distinctions in their relative performance. A common approach to compare methods is to complete multiple assessments of the same fish assemblage using different methods. Many such methodological comparisons exist in the literature (e.g., [24], [26], [34], [35], [29], [36], [37], [38]), providing insights into the pairwise similarities and differences in results generated from competing methods. Importantly, these comparisons evaluate relative performance (e.g., method *x* consistently estimates a higher density of large-bodied species relative to method *y*) instead of true performance (e.g., method *z* overestimates the density of large-bodied species relative to the true density). This is because in few cases do we have an unbiased estimate of the ‘true’ density of any fish species to serve as a rigorous benchmark. The only exceptions are the small-bodied, sedentary species (e.g., gobies, blennies) that can be sampled effectively through extractive sampling such as the use of rotenone or other ichthyocides [39], [40]. Given that the application of *in situ* reef fish assessments is often aimed at larger-bodied taxa, such as those of importance for reef fisheries, our evaluations of methodological performance offer limited insights into ‘true’ performance; in few cases are results of relative performance clear-cut (e.g., one method wholly misses quantification of particular taxa). In most cases, instead, users are required to decide on methods based upon relative performance and their own expert judgment regarding the strengths and weaknesses of the methods available.

Even though no assessment method for reef fishes can be deemed ‘correct’ based upon an unbiased metric of true performance, there remain a number of data applications that depend upon methodological consistency to generate meaningful results. A growing number of studies aim to evaluate the similarities and differences in the composition of reef fish assemblages, in some cases reporting potential changes through time (e.g., after establishment of a no-take protected area) [41,42]; long-term trends [43]; or variation across locations (e.g., across sites with differing fishing pressure) [14,15,17] or differing environmental conditions [44–46]. The strength of such studies is founded on the reliability of comparisons across datasets. Variation in survey methodology can decrease direct comparability across datasets, potentially increase sampling error, and in the worst case introduce fatal sampling biases and incorrect results.

This study was designed to increase our understanding of the patterns and motivations of researchers working on reef fishes in their selection of particular assessment methods. We conducted a survey of researchers from across the globe to determine which method (or methods) individuals’ use and the driving forces behind their particular choice. Further, we asked whether there was motivation to standardize sampling methods, and if so, what were the challenges to realizing a global standard. This study was conducted to identify drivers of method selection, enhance comparability of future research efforts, and ultimately facilitate improved management of nearshore fish assemblages.

## Materials and Methods

The University of California at San Diego Institutional Review Board approved this study, Project number: 110298SX.

### Questionnaire design

A broad sociological survey was conducted in order to determine what drivers influence the selection of *in situ* fish census method by members of the research community. The data were collected using the online survey platform, the Qualtrics Research Suite, Version 37, 892 (Qualtrics, Provo, UT). The survey was split into two sections. The first section collected data on methods currently being used to assess reef fish assemblages and diversity. The second section collected thoughts and input from the research community on the issue of developing and implementing a standardized fish surveying method for reef fishes. The entire survey is presented in S1 and S2 Files.

### Identification of Respondents

Respondents to the survey were identified through research directories, listserves, and through chain referral sampling from other respondents. An initial list of researchers was gathered from existing colleagues and contacts in academic, government, and non-government organizations worldwide. Further, NOAA’s CORAL-List (www.coral.aoml.noaa.gov/mailman/listinfo/coral-list) and the American Academy of Underwater Sciences (www.aaus.org) directories were used as forums to reach a large part of the research community. Upon identifying a respondent, an e-mail was sent providing background information about the survey and including a link to the survey questionnaire. Included in the e-mail was an option for the respondent to forward the questionnaire on to colleagues. As results were analyzed, a summary map was generated iteratively to identify where responses had been gathered. This map was sent back to the original email list and forums in hopes of generating regionally-based incentive to gather information from respondents in regions that were underrepresented during each iteration of the summary map. A regionally-based search for relevant researchers was conducted internally to gather information from regions that were lacking responses.

It was recorded that many individuals were involved in more than one research project, sometimes employing different methods. To account for this, each unique combination of respondent and research project was included as an individual replicate.

### Location of Respondents’ Home Institution

**Fig 1:**
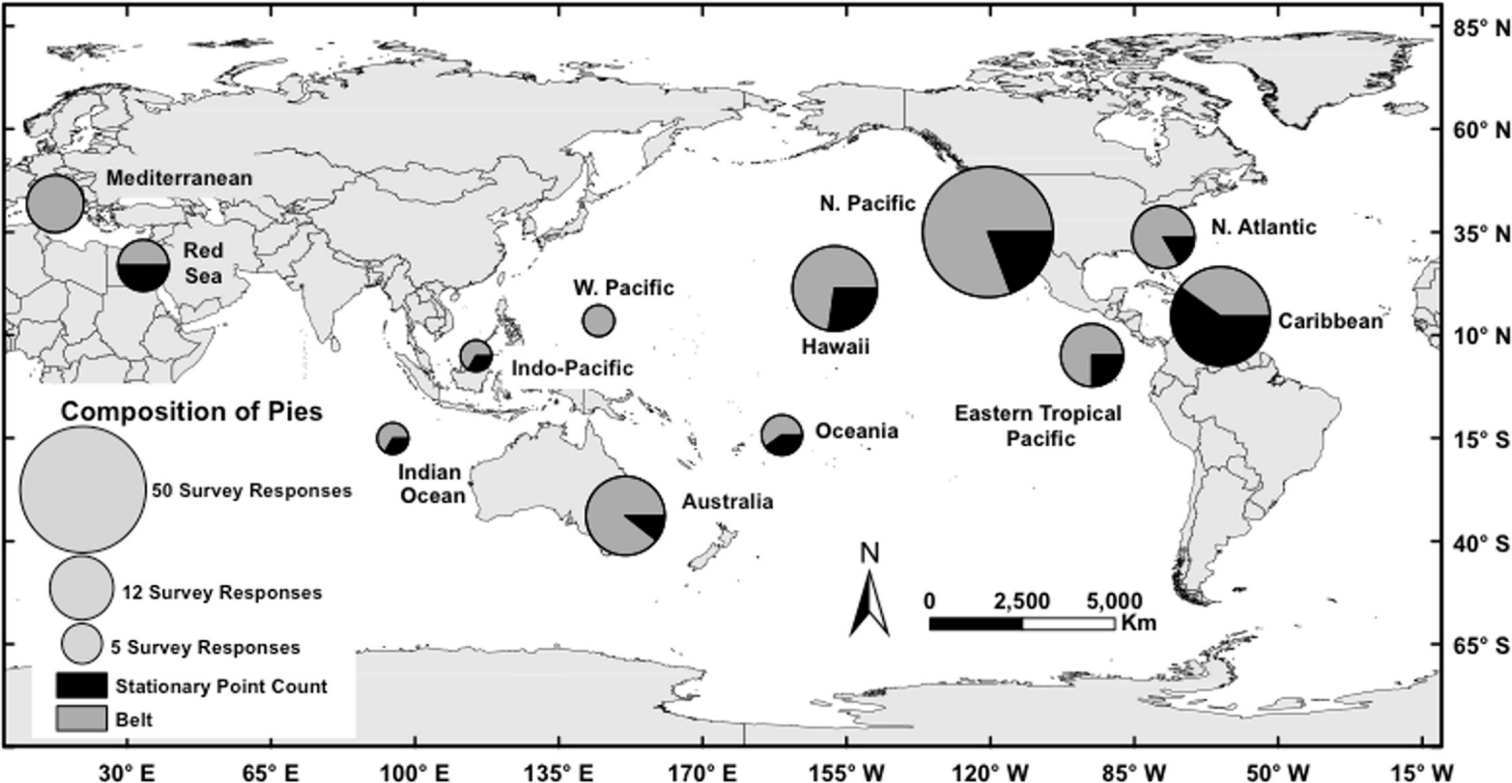
Relative utilization of the two most dominant underwater visual census (UVC) methods (stationary point count versus belt transect) by survey region. The size of the circle corresponds to the number of projects reported on from each study region, with the proportion of each method contributing to this overall total (n = 298) shown.

**Fig 2:**
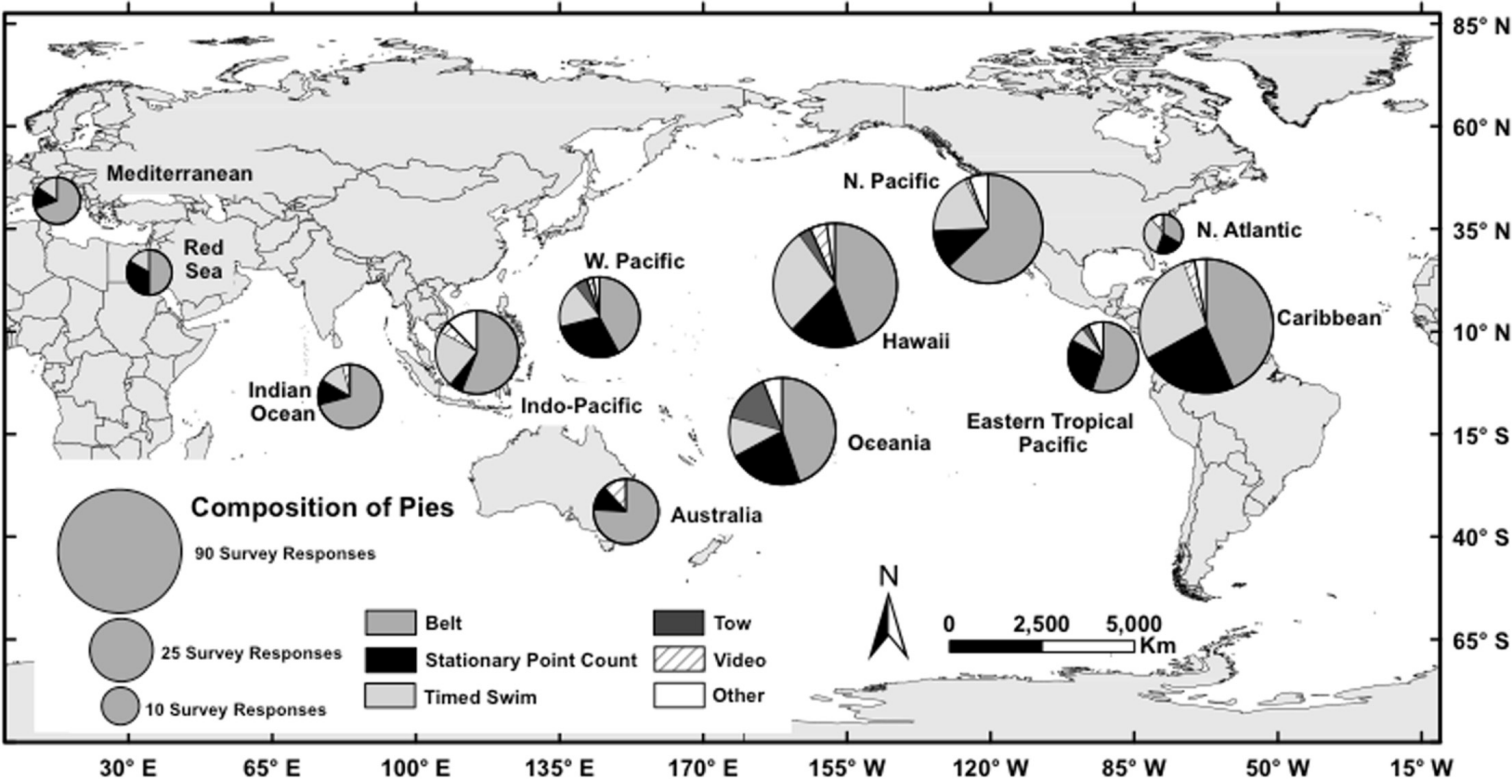
Global distribution of underwater visual census (UVC) methods used to quantify reef fish assemblages. The size of the circle corresponds to the number of projects reported on from each study region, with the proportion of each method contributing to the overall total (n = 426) shown.

To determine the location of each respondent, we relied on the unique Internet Protocol (IP) address associated with each completed survey. An IP address is a unique numerical label assigned to computers or other hardware that identifies the location where a computer is connected to the network or Internet. We used an IP locator tool to identify the respondents’ country and probable home institution. Visualizations of the responses were created using ArcGIS software [47] to show the geographic distribution of the reported survey-based projects (Figs 1 and 2).

### Statistical analyses

Analyses were performed to test the dependence of method used on the following drivers: study location, study duration, research question being asked, and researcher location. We also tested for the possible link between respondent’s willingness to modify methodologies to a standardized method versus the motivation for the study, the question being asked, and the duration of the study. A permutational Pearson's Chi-squared test (based on 9999 permutations of the raw data) was used to determine statistical significance. All analyses were conducted using R 2.15.3 (R Development Core Team, www.r-project.org).

## Results

It is estimated that the email with the survey link attached reached at least 4000 individuals in the research community. It is understood that some of these email recipients were not in the field of assessing reef fish assemblages but may have had expertise in other areas of marine science. These individuals would not have an interest or incentive to open the email or conduct the survey. It was calculated that 417 of those researchers who received the survey actually opened the link and started the survey. 180 individuals submitted completed surveys. The methods recorded represented 191 countries, islands, regions, states, and communities. Of the survey-based projects reported, 49.8% employed the belt transect method, 20.2% used the stationary point count method, and 19.0% of projects used some variation of a timed swim method (Table 2). The timed swim was reported almost as frequently as the stationary point count method but was usually used in conjunction with another survey method and largely seemed to focus on larger bodied fishes. Belt transect and stationary point count were the most widely used methods to assess fish assemblages as recorded in this study, and seem to be associated with the most arguments regarding method efficacy [24]. We therefore investigated these two particular methods in detail.

**Table 2:** Underwater visual census (UVC) methods used to quantify reef fish assemblages. The count per method is enumerated for the entire 426 projects reported on by the 180 survey respondents. Note that the five most commonly reported methods represent over 90% of the total responses. ‘Other’ category includes all rarely employed methods noted by only one respondent.

Method	Number
Belt	212
Stationary Point Count	86
Timed Swim	81
Video Survey	18
Towed Diver Survey	11
Other	18

Researchers’ choice of survey method was found to be independent of the study location (χ^2^ = 3.05, p = 0.70), independent of the duration of the study (χ^2^ = 2.46, p = 0.69), and independent of the research question being asked (χ^2^ = 6.02, p = 0.97). However, choice of methodology was found to be influenced by the region where the researcher is based (as determined by IP address; χ^2^ = 11.3, p = 0.04). Researchers based in Florida and the Caribbean disproportionately reported use of the stationary point count method while researchers based in Australia, Hawaii, and the eastern Pacific overwhelmingly reported use of the belt transect method (Fig 1). The willingness to modify methodologies to a standardized method was found to be independent of the motivation for the study (χ^2^ = 5.65, p = 0.47), and independent of the question being asked (χ^2^ = 7.75, p = 0.92). The professional role of the individual, however, was significant as researchers in managerial roles were more likely to adopt or modify their protocols to produce comparable data than researchers in academia (χ^2^ = 4.58, p = 0.04).

The majority of researchers surveyed (80%) said that they see a potential benefit in having a standardized method that would produce comparable data across regions. However, researchers’ willingness to modify their methodology was dependent on the duration of their project (χ^2^ = 13.16, p = 0.01); researchers with longer-term datasets of five or more years were less willing to modify their methods than were those with shorter-term or single snapshot datasets. The foremost reason researchers expressed hesitation to adopt a standardized method was the fear of long-term datasets or previous monitoring efforts becoming incomparable or obsolete.

## Discussion

*In situ* assessments of reef fish assemblages are a common tool for the research community, and can aid in addressing ecological questions and informing management decisions. The literature is replete with examples of survey methodologies, which span a wide range of intended purposes and applications (Table 1). While the variety of methods provides options for the research community, this variety can also lead to challenges, particularly given the growing interest in spatio-temporal assessments of reef fish assemblages. In order to achieve a goal of intercomparability of fish assemblage structure, it will be valuable to standardize sampling approaches in order to minimize the error associated with methodological variability. The issue of method standardization is not limited to reef surveys. The importance of standardizing methodologies in hopes of providing resource managers with comprehensive datasets has proven important in terrestrially-based research as well. For example, foresters commonly measure the diameter of a tree at breast height to estimate tree height and biomass. This method reduces the opportunity for error in calculations and produces very accurate and comparable information when assessing biomass in timber stands for many tree species across regions [48–50].

Based on our survey of researchers working with reef fish, we show evidence that a limited number of *in situ* survey methods are used commonly. In particular, we found that 89% of the survey-based projects reported were conducted using one of three methods–belt transect, stationary point count, and a variant of the timed swim (Table 2, Fig 2). The relative popularity and commonness of these methods has been reported by recent reviews of the efficacy of observational approaches for quantifying reef fishes [25]. It is important to note, however, that the results presented here are based upon a voluntary survey of researchers, and despite our efforts to achieve broad geographic representation of respondents, it is possible that these estimates of relative rates of use among methods is not fully representative of those used globally. One example of potential under-representation is the relative paucity of respondents reporting use of distance sampling (Tables 1 and 2). Despite some potential for bias in the representation of methods being used and users themselves in this survey, we are encouraged with the concordance of our survey results and the conclusions of published reviews of reef fish enumeration studies [25]; both approaches reveal a relatively small number of methods representing the majority of survey-based projects.

Based on a broad survey of the research community, we found evidence of a general consensus on the value of standardizing methods for surveying reef fish assemblages, as 80% of researchers surveyed agreed that there was a research benefit to standardizing methodologies. Despite such evidence of growing consensus, strong divisions remain in the community that appear to prevent more generalized agreement in field approaches. Our study identified two principal hurdles that challenge the realization of methodological standardization in reef fish assessment. First, a researcher’s geographical origin appears to strongly dictate the choice of method used. Second, a researcher’s willingness to modify their existing method was dependent on the duration of their study, with those researchers associated with long-term datasets less willing to change a currently employed method and adopt a different, though perhaps standardized, method.

A major factor influencing a researcher’s choice in method was their geographical origin. For example, the stationary point count method predominated in institutions located in Florida and the Caribbean while the belt transect method was widely used by institutions across Australia, Hawaii, and the Eastern Pacific. Notably, this association was based on the location of the respondent’s home institution, and was distinct from the field site in which the methodology was employed. As such, this finding suggests that choice of methodology is less related to physical or environmental constraints within a study site and more associated with academic tradition or region-specific training. Although conclusions about the causality of this association are beyond the scope of this survey, results suggest that home region may be a driving influence in choice of methodology and may prove to be a barrier to efforts at standardization.

Our understanding of the broad ecological effects of humans on nearshore ecosystems has advanced as a result of consistent monitoring programs sampling large geographic areas [15,17–19,51] over long time periods [41–43,52,53]. Researchers involved in these programs are understandably hesitant to alter sampling methods. Our survey revealed that researchers with large spatial or temporal datasets were less willing to change to a standardized method due to the fear of historical datasets becoming incomparable or obsolete. This presents an interesting conundrum–how can we ensure long-term datasets are not compromised while increasing comparability across studies into the future? One response would be to develop a reliable approach for data conversion across methods. Similar conversions have been developed and used in fisheries research when standardizing CPUE data that are gathered using multiple gear types [54]. One solution could be a broad methodological database of the most common assessment methods (e.g., belt transect and stationary point count), with paired surveys conducted in many geographies and environmental conditions. If such a database were publicly available, a statistically robust conversion protocol could be established. Thus, the integrity of long-term or large-area monitoring programs could be maintained, while the results from multiple programs employing different methods could be compared rigorously. Importantly, such an approach is only valid if the error of conversion is incorporated into the final estimates, highlighting the primary point that methodological consistency, when possible, provides the most powerful approach for comparing fish assemblages.

While the idea of adopting a single authoritative methodology for all research and all regions may be neither practical nor desirable, standardization does not require a single monolithic approach. In addition to the data conversion approach mentioned above, a standardized decision tree leading to a suite of appropriate survey methods may also contribute toward standardization of research with similar questions or in similar systems. Work has been published that highlights and compares common survey methodologies for various systems and fields [55–57], which could contribute to the production of such a tool.

The focus of this survey was to gather information and perspectives on standardization from researchers already using visual census methodologies for basic and applied research across the globe. An additional critical component, however, is the comparability of future research and the benefits that broad comparison may confer in the context of global environmental change. One of our main findings was that choice of methodology does not show a statistically significant association with either research question or ecological system, but is most associated with epistemological or institutional attributes. This suggests that individuals in the research community should be cautious of choosing a survey method based solely on local influence, as it may not be the most beneficial for successful large-scale management, conservation, or scientific comparison. Frequently, the primary goal of fisheries surveys is increasing understanding of, and bettering, the health of our ocean’s resources. One of the most effective ways of achieving this goal is to provide scientists, managers, and policymakers with the most complete and comparable datasets possible; this may ensure that managers can measure the impacts of certain activities, compare these effects, and make the most effective and educated management decisions possible. Developing and implementing standardized survey methodologies that are effective across regions is one way to provide managers with that information.

For researchers aiming to understand the structure of reef fish assemblages, many potential methods are available. The selection of method will be based upon a suite of information, ranging from relative performance estimates through to operational constraints. Looking toward the future, there are countless opportunities to track geographic and temporal patterns in the structure of reef fish assemblages. Despite a strong community of taxonomically-savvy researchers, however, methodological inconsistency will limit the potential for comparisons of data at grand scales, and thus limit the applicability of visual census surveys. This study provides insights into some of the challenges and opportunities available for increased methodological consistency. Awareness of comparability in initial methodology selection, as well as stronger focus towards communication and partnerships on a regional basis, are critical in order for the community to overcome some of these challenges. The problems facing reef fish populations are persistent and global, and therefore require a global and cooperative approach to monitoring and assessment—standardized methodology design represents an important step in broadening our ability to understand and address these problems. The data presented in this paper as well as other published works can act as important tools in choosing a method that is most comparable with the existing and growing datasets regionally and globally.

## Supporting Information

S1 File Online Survey. (DOCX)

S2 File List of Survey Questions Included in the Survey. (DOCX)

S3 File Description of Top Three Survey Methodologies. (DOCX)

## References

[1] Tissot BN, Hallacher LE. Effects of Aquarium Collectors on Coral Reef Fishes in Kona, Hawaii. Conservation Biology. 2003;17: 1759–1768. 10.1111/j.1523-1739.2003.00379.x

[2] Knowlton N, Jackson JBC. Shifting Baselines, Local Impacts, and Global Change on Coral Reefs. PLOS Biol. 2008;6: e54 10.1371/journal.pbio.0060054 18303956 PMC2253644

[3] Munday PL, Jones GP, Pratchett MS, Williams AJ. Climate change and the future for coral reef fishes. Fish and Fisheries. 2008;9: 261–285. 10.1111/j.1467-2979.2008.00281.x

[4] Jackson JB, Kirby MX, Berger WH, Bjorndal KA, Botsford LW, Bourque BJ, et al Historical overfishing and the recent collapse of coastal ecosystems. Science. American Association for the Advancement of Science; 2001;293: 629–637. 10.1126/science.1059199 11474098

[5] Dulvy NK, Freckleton RP, Polunin NVC. Coral reef cascades and the indirect effects of predator removal by exploitation. Ecol Lett. Blackwell Science Ltd; 2004;7: 410–416. 10.1111/j.1461-0248.2004.00593.x

[6] Pandolfi JM, Bradbury RH, Sala E, Hughes TP, Bjorndal KA, Cooke RG, et al Global trajectories of the long-term decline of coral reef ecosystems. Science. American Association for the Advancement of Science; 2003;301: 955–958. 10.1126/science.1085706 12920296

[7] Johnson AE. Reducing bycatch in coral reef trap fisheries: escape gaps as a step towards sustainability. Mar Ecol Prog Ser. 2010;415: 201–209. 10.3354/meps08762

[8] Hawkins JP, Roberts CM, Gell FR, Dytham C. Effects of trap fishing on reef fish communities. Aquatic Conservation: Marine and Freshwater Ecosystems. 2007;17: 111–132. 10.1002/aqc.784

[9] Bell JD, Craik GJS, Pollard DA, Russell BC. Estimating length frequency distributions of large reef fish underwater. Coral Reefs. 1985;4: 41–44. 10.1007/BF00302203

[10] Jennings S, Kaiser MJ. The Effects of Fishing on Marine Ecosystems. Elsevier; 1998 pp. 201–352. 10.1016/S0065-2881(08)60212-6

[11] Jennings SPD, Kaiser MJ, Reynolds JDPD. Marine fisheries ecology Oxford; Malden, MA, USA: Blackwell Science; 2001.

[12] King M. Fisheries Management Fisheries Biology, Assessment and Management. Oxford, UK: Blackwell Publishing Ltd; 2013 pp. 273–315. 10.1002/9781118688038.ch6

[13] Brock VE. A Preliminary Report on a Method of Estimating Reef Fish Populations. The Journal of Wildlife Management. 1954;18: 297 10.2307/3797016

[14] DeMartini EE, Friedlander AM, Sandin SA, Sala E. Differences in fish-assemblage structure between fished and unfished atolls in the northern Line Islands, central Pacific. Mar Ecol Prog Ser. 2008;365: 199–215. 10.3354/meps07501

[15] Friedlander AM, DeMartini EE. Contrasts in density, size, and biomass of reef fishes between the northwestern and the main Hawaiian islands: the effects of fishing down apex predators. Mar Ecol Prog Ser. 2002;230: 253–264. 10.3354/meps230253

[16] Hamilton SL, Caselle JE, Malone DP, Carr MH. Incorporating biogeography into evaluations of the Channel Islands marine reserve network. Proceedings of the National Academy of Sciences. 2010;107: 18272–18277. 10.1073/pnas.0908091107 PMC2973008 20176956

[17] Sandin SA, Smith JE, DeMartini EE, Dinsdale EA, Donner SD, Friedlander AM, et al Baselines and Degradation of Coral Reefs in the Northern Line Islands. Ahmed N, editor. PLOS ONE. 2008;3: e1548 10.1371/journal.pone.0001548 18301734 PMC2244711

[18] Stallings CD. Fishery-Independent Data Reveal Negative Effect of Human Population Density on Caribbean Predatory Fish Communities. Bruno JF, editor. PLOS ONE. 2009;4: e5333 10.1371/journal.pone.0005333 19421312 PMC2672166

[19] Williams ID, Richards BL, Sandin SA, Baum JK, Schroeder RE, Nadon MO, et al Differences in Reef Fish Assemblages between Populated and Remote Reefs Spanning Multiple Archipelagos Across the Central and Western Pacific. Journal of Marine Biology. 2011;2011: 1–14. 10.1155/2011/826234

[20] Zgliczynski BJ, Williams ID, Schroeder RE, Nadon MO, Richards BL, Sandin SA. The IUCN Red List of Threatened Species: an assessment of coral reef fishes in the US Pacific Islands. Coral Reefs. 2013;32: 637–650. 10.1007/s00338-013-1018-0

[21] Bohnsack JA, Bannerot SP. A Stationary Visual Census Technique for Quantitatively Assessing Community Structure of Coral Reef Fishes. 1986.

[22] Bellwood DR, Alcala AC. The effect of a minimum length specification on visual estimates of density and biomass of coral reef fishes. Coral Reefs. 1988;7: 23–27. 10.1007/BF00301978

[23] Helfman G, Collette BB, Facey DE, Bowen BW. The Diversity of Fishes. John Wiley & Sons; 2009.

[24] Samoilys MA, Carlos G. Determining Methods of Underwater Visual Census for Estimating the Abundance of Coral Reef Fishes. Environ Biol Fish. Kluwer Academic Publishers; 2000;57: 289–304. 10.1023/A:1007679109359

[25] Usseglio P. Quantifying reef fishes: bias in observational approaches In: Mora C, editor. Ecology of Fishes on Coral Reefs. Cambridge: Cambridge University Press; 2015 pp. 270–273. 10.1017/CBO9781316105412.035

[26] Brock RE. A Critique of the Visual Census Method for Assessing Coral Reef Fish Populations. University of Miami—Rosenstiel School of Marine and Atmospheric Science; 1982.

[27] Lindfield SJ, Harvey ES, McIlwain JL, Halford AR. Silent fish surveys: bubble-free diving highlights inaccuracies associated with SCUBA-based surveys in heavily fished areas. Börger L, editor. Methods Ecol Evol. 2014;5: 1061–1069. 10.1111/2041-210X.12262

[28] Feary DA, Cinner JE, Graham NAJ, Januchowski-Hartley FA. Effects of Customary Marine Closures on Fish Behavior, Spear-Fishing Success, and Underwater Visual Surveys. Conservation Biology. 2010;: no–no. 10.1111/j.1523-1739.2010.01613.x 21129032

[29] Kulbicki M. How the acquired behaviour of commercial reef fishes may influence the results obtained from visual censuses. Journal of Experimental Marine Biology and Ecology. 1998;222: 11–30. 10.1016/S0022-0981(97)00133-0

[30] Nevill J. The impacts of spearfishing: notes on the effects of recreational diving on shallow marine reefs in southern Australia. OnlyOnePlanet Australia. 2005.

[31] Chapman CJ, Johnstone ADF, Dunn JR, Creasey DJ. Reactions of fish to sound generated by divers' open-circuit underwater breathing apparatus. Mar Biol. 1974;27: 357–366. 10.1007/BF00394372

[32] Rizzari JR, Frisch AJ, Connolly SR. How robust are estimates of coral reef shark depletion? Biological Conservation. 2014;176: 39–47. 10.1016/j.biocon.2014.05.003

[33] Bozec Y-M, Kulbicki M, Laloë F, Mou-Tham G, Gascuel D. Factors affecting the detection distances of reef fish: implications for visual counts. Mar Biol. 2011;158: 969–981. 10.1007/s00227-011-1623-9

[34] Mapstone BD, Ayling T. An Investigation of Optimum Methods and Unit Sizes for the Visual Estimation of Abundances of Some Coral Reef Organisms. 1998.

[35] McCauley DJ, McLean KA, Bauer J, Young HS, Micheli F. Evaluating the performance of methods for estimating the abundance of rapidly declining coastal shark populations. Ecological Applications. 2012;22: 385–392. 10.1890/11-1059.1 22611841

[36] Kulbicki M. Comparison of density estimates derived from strip transect and distance sampling for underwater visual censuses: a case study of Chaetodontidae and Pomacanthidae. Aquatic Living Resources. 1999;12: 315–325. 10.1016/S0990-7440(99)00116-3

[37] Colvocoresses J, Acosta A. A large-scale field comparison of strip transect and stationary point count methods for conducting length-based underwater visual surveys of reef fish populations. Fisheries Research. 2007;85: 130–141. 10.1016/j.fishres.2007.01.012

[38] Kulbicki M, Cornuet N, Vigliola L, Wantiez L, Moutham G, Chabanet P. Counting coral reef fishes: Interaction between fish life-history traits and transect design. Journal of Experimental Marine Biology and Ecology. 2010;387: 15–23. 10.1016/j.jembe.2010.03.003

[39] Willis TJ, Anderson MJ. Structure of cryptic reef fish assemblages: relationships with habitat characteristics and predator density. Mar Ecol Prog Ser. 2003;257: 209–221. 10.3354/meps257209

[40] Ackerman JL, Bellwood DR. Reef fish assemblages: a re-evaluation using enclosed rotenone stations. Mar Ecol Prog Ser. 2000;206: 227–237. 10.3354/meps206227

[41] Wantiez L, Thollot P, Kulbicki M. Effects of marine reserves on coral reef fish communities from five islands in New Caledonia. Coral Reefs. 1997;16: 215–224. 10.1007/s003380050077

[42] McClanahan TR, Graham N. Recovery trajectories of coral reef fish assemblages within Kenyan marine protected areas. Mar Ecol Prog Ser. 2005;294: 241–248. 10.3354/meps294241

[43] Halford A, Cheal AJ, Ryan D, Williams DM. Resilience to large-scale disturbance in coral and fish assemblages on the Great Barrier Reef. Ecology. 2004;85: 1892–1905. 10.1890/03-4017

[44] Edgar GJ, Barrett NS, Morton AJ. Biases associated with the use of underwater visual census techniques to quantify the density and size-structure of fish populations. Journal of Experimental Marine Biology and Ecology. 2004;308: 269–290. 10.1016/j.jembe.2004.03.004

[45] Williams ID, Baum JK, Heenan A, Hanson KM, Nadon MO, Brainard RE. Human, Oceanographic and Habitat Drivers of Central and Western Pacific Coral Reef Fish Assemblages. Ferse SCA, editor. PLOS ONE. 2015;10: e0120516 10.1371/journal.pone.0120516 25831196 PMC4382026

[46] Richards BL, Williams ID, Vetter OJ, Williams GJ. Environmental Factors Affecting Large-Bodied Coral Reef Fish Assemblages in the Mariana Archipelago. Thrush S, editor. PLOS ONE. 2012;7: e31374 10.1371/journal.pone.0031374 22384014 PMC3288046

[47] ArcGIS. 10 ed. Redlands, CA: ESRI, Environmental Systems Research Institute, Inc.

[48] Colbert K, Larsen DR. Height-diameter Equations and Mortality Rates for Thirteen Midwest Bottomland Hardwood Species. Northern Journal of Applied Forestry. 2002;19: 171–176.

[49] Curtis RO. Height-Diameter and Height-Diameter-Age Equations For Second-Growth Douglas-Fir. Society of American Foresters; 1967.

[50] Larsen DR, Hann DW, Oregon State University. Forest Research Laboratory. Height-diameter equations for seventeen tree species in southwest Oregon. Corvallis, OR: Forest Research Laboratory, College of Forestry, Oregon State University; 1987.

[51] Stuart-Smith RD, Bates AE, Lefcheck JS, Duffy JE, Baker SC, Thomson RJ, et al Integrating abundance and functional traits reveals new global hotspots of fish diversity. Nature. 2013;501: 539–542. 10.1038/nature12529 24067714

[52] Heenan A, Gorospe K, Williams ID, Levine A, Maurin P, Nadon M, et al Ecosystem monitoring for ecosystem-based management: Using a polycentric approach to balance information trade-offs. J Appl Ecol. 2016;: n/a–n/a. 10.1111/1365-2664.12633

[53] Paddack MJ, Reynolds JD, Aguilar C, Appeldoorn RS, Beets J, Burkett EW, et al Recent Region-wide Declines in Caribbean Reef Fish Abundance. Curr Biol. 2009;19: 590–595. 10.1016/j.cub.2009.02.041 19303296

[54] Cheung WWL, Pitcher TJ. Evaluating the status of exploited taxa in the northern South China Sea using intrinsic vulnerability and spatially explicit catch-per-unit-effort data. Fisheries Research. 2008;92: 28–40. 10.1016/j.fishres.2007.12.018

[55] Hill J, Wilkinson C. Methods for ecological monitoring of coral reefs Australian Institute of Marine Science 2004.

[56] Barea-Azcón JM, Virgós E, Ballesteros-Duperón E, Moleón M, Chirosa M. Surveying carnivores at large spatial scales: a comparison of four broad-applied methods. Biodivers Conserv. 2006;16: 1213–1230. 10.1007/s10531-006-9114-x

[57] Bibby C, Jones M, Marsden S. Expedition field techniques: bird surveys. Royal Geographical Society; 1998.

[58] Branden KL, Shepherd SA, Edgar GJ. Reef fish populations of the Investigator Group [islands], South Australia: a comparison of two census methods. Transactions of the Royal Society of South Australia (Australia). 1986.

[59] Thresher RE, Gunn JS. Comparative analysis of visual census techniques for highly mobile, reef-associated piscivores (Carangidae). Environ Biol Fish. 1986;17: 93–116. 10.1007/BF00001740

[60] Willis TJ. Visual census methods underestimate density and diversity of cryptic reef fishes. Journal of Fish Biology. 2001;59: 1408–1411. 10.1111/j.1095-8649.2001.tb00202.x

[61] Guidetti P, Bussotti S, Boero F. Evaluating the effects of protection on fish predators and sea urchins in shallow artificial rocky habitats: a case study in the northern Adriatic Sea. Marine Environmental Research. 2005;59: 333–348. 10.1016/j.marenvres.2004.05.008 15589985

[62] Minte-Vera CV, de Moura RL, Francini-Filho RB. Nested sampling: an improved visual-census technique for studying reef fish assemblages. Mar Ecol Prog Ser. 2008;367: 283–293. 10.3354/meps07511

[63] Dickens LC, Goatley CHR, Tanner JK, Bellwood DR. Quantifying Relative Diver Effects in Underwater Visual Censuses. Unsworth RKF, editor. PLOS ONE. 2011;6: e18965 10.1371/journal.pone.0018965 21533039 PMC3080881

[64] Menza CW, Ault JS, Beets J, Bohnsack JA, Caldow C, Christensen J, et al A guide to monitoring reef fish in the National Park Service's South Florida/Caribbean Network NOAA/National Ocean Service; 2006.

[65] Halford AR, Thompson AA. Visual Census Surveys of Reef Fish Longterm Monitoring of the Great Barrier Reef Standard Operational Procedure Number 3. Visual Census Surveys of Reef Fish Australian Institute …; 1994.

[66] Smith SG, Ault JS, Bohnsack JA, Harper DE, Luo J, McClellan DB. Multispecies survey design for assessing reef-fish stocks, spatially explicit management performance, and ecosystem condition. Fisheries Research. 2011;109: 25–41. 10.1016/j.fishres.2011.01.012

[67] Bohnsack JA, McClellan DB, Harper DE, Davenport GS. Baseline data for evaluating reef fish populations in the Florida Keys, 1979–1998. 1999.

[68] Robbins WD, Hisano M, Connolly SR, Choat JH. Ongoing collapse of coral-reef shark populations. Curr Biol. 2006;16: 2314–2319. 10.1016/j.cub.2006.09.044 17141612

[69] Jones RS, Thomson MJ. Comparisons of Florida reef fish assemblages using a rapid visual technique. BMS. 1978;28: 179–172.

[70] Pattengill-Semmens CV, Semmens BX. Fish census data generated by non-experts in the Flower Garden Banks National Marine Sanctuary. Journal of Gulf of Mexico Science. 1998.

[71] Richards BL, Williams ID, Nadon MO, Zgliczynski BJ. A towed-diver survey method for mesoscale fishery-independent assessment of large-bodied reef fishes. BMS. 2011;87: 55–74. 10.5343/bms.2010.1019

[72] Holzwarth SR, DeMartini EE, Schroeder RE. Sharks and jacks in the Northwestern Hawaiian Islands from towed-diver surveys 2000–2003. Atoll Res …. 2006.

[73] Done TJ. Rapid, large area, reef resource surveys using a manta board. 1981.

[74] Harvey E, Fletcher D, Shortis MR, Kendrick GA. A comparison of underwater visual distance estimates made by scuba divers and a stereo-video system: implications for underwater visual census of reef fish abundance. Mar Freshwater Res. 2004;55: 573 10.1071/MF03130

[75] Watson DL, Harvey ES, Anderson MJ, Kendrick GA. A comparison of temperate reef fish assemblages recorded by three underwater stereo-video techniques. Mar Biol. 2005;148: 415–425. 10.1007/s00227-005-0090-6

[76] Colton MA, Swearer SE. A comparison of two survey methods: differences between underwater visual census and baited remote underwater video. Mar Ecol Prog Ser. 2010;400: 19–36. 10.3354/meps08377

[77] Langlois TJ, Harvey ES, Fitzpatrick B, Meeuwig JJ, Shedrawi G, Watson DL. Cost-efficient sampling of fish assemblages: comparison of baited video stations and diver video transects. Aquat Biol. 2010;9: 155–168. 10.3354/ab00235

[78] Watson DL, Harvey ES, Fitzpatrick BM, Langlois TJ, Shedrawi G. Assessing reef fish assemblage structure: how do different stereo-video techniques compare? Mar Biol. 2010;157: 1237–1250. 10.1007/s00227-010-1404-x

[79] Mallet D, Pelletier D. Underwater video techniques for observing coastal marine biodiversity: A review of sixty years of publications (1952–2012). Fisheries Research. 2014;154: 44–62. 10.1016/j.fishres.2014.01.019

[80] Willis TJ, Babcock RC. A baited underwater video system for the determination of relative density of carnivorous reef fish. Mar Freshwater Res. CSIRO PUBLISHING; 2000;51: 755–763. 10.1071/MF00010

[81] Cappo M, Harvey E, Malcolm H. Potential of video techniques to monitor diversity, abundance and size of fish in studies of marine protected areas. Aquatic Protected Areas-2003.

[82] Kenyon JC, Brainard RE, Hoeke RK, Parrish FA, Wilkinson CB. Towed-Diver Surveys, a Method for Mesoscale Spatial Assessment of Benthic Reef Habitat: A Case Study at Midway Atoll in the Hawaiian Archipelago. Coastal Management. 2006;34: 339–349. 10.1080/08920750600686711

