# Supplementary material for: Reef Fish Survey Techniques: Assessing the Potential for Standardizing Methodologies
Source: PLoS One. 2016 Apr 25;11(4):e0153066. doi: 10.1371/journal.pone.0153066 (PMC4844186; doi:10.1371/journal.pone.0153066)
Supplement: S1 File — (DOCX) [file pone.0153066.s001.docx]

**S1 File**

S1. Online survey

My name is Zach Caldwell and I am a Masters of Advanced Studies student through the Center for Marine Biodiversity and Conservation program at the University of California at San Diego hosted by Scripps Institution of Oceanography.

I am conducting research that aims at better understanding where certain reef fish census methods are being used. I would also like to collect information that helps us all understand why a standardized survey method has not been developed and get a better feeling of how important this topic is to the research community. You have been selected as a participant in this survey because of your experience in this field. The following survey will take roughly ten minutes. Your participation in this project by completing this short survey is very much appreciated. Thank you so much for your time.

1. Where is this research involving fish surveys currently being conducted? (Please specify site name, country, body of water, etc.)

Write in answer…

2. What is the general research question that these fish surveys will help to answer for this research?

Write in answer…

3. What is the purpose of data you are collecting? (Check all that apply)

- 1. For long term monitoring
  2. In house research
  3. Masters or PhD thesis
  4. Drive management decisions
  5. Assess long term effectiveness of new management decisions
  6. Rapid assessment or short term project
  7. For an initial baseline survey
  8. To test efficacy of management
  9. Other. Please specify…

4. What survey method is being used in your research? Check all that apply

a. Belt transect

b. Stationary Point Count

c. Timed swim

1. Other. Please specify

5. Please provide a detailed description of the dimensions of your protocols. . (feel free to add a description of the methodology from a paper that describes the methods)

6. How did you choose the methods for the project? (Check all that apply)

a. Produces data that are most comparable to other research institutions or projects

b. To assure consistency with historical data

b. Best fit for the research questions being asked

c. Worked best logistically with the resources at hand

1. Only method learned
2. Other please specify

7. How were these methods developed?

a. Designed by yourself or within your home institution

b. Adopted from another research institution or project

c. Adopted and **modified** from another research institution

e. Other. Please specify

8. How long have you been conducting your research for this project using this method?

1. 0-2 years
2. 2-5 years
3. 5-10 years
4. 10 years or more

9. If data are collected through time, how often do you conduct surveys for **this** project using **these** methods?

1. Weekly
2. biweekly
3. Monthly
4. bimonthly
5. Semi-annually
6. Annually
7. Other, please specify…

10. How long do you plan to continue this research project?

Write in answer…

Section 2

A variety of methods are currently being used around the globe to measure and estimate reef fish populations. Discussion has been ongoing as to the importance of developing a standardized reef fish census method that could be used across regions and produce datasets that would be comparable to one another. The following questions are aimed to gather information as to your thoughts on standardizing reef fish census methodology.

11. Do you think it is valuable to have a single method across regions?

1. Yes, please explain…

b. No, please explain…

12. To what extent are you willing to modify your current methodology to produce a standardized reef fish survey method?

1 2 3 4 5 6 7 8 9 10

**Not willing to change Adopt new methods**

13. What factors would increase your interest to adopt a new survey methodology?

a. If the majority of colleagues changed as well

b. Made data more comparable to other large datasets

c. Ensure that existing data would not become irrelevant

d. If standardized method continued to answer the existing research questions of the project

e. Other please specify….

14. Why do you believe there has not been one standardized fish survey method adopted across all regions?

a. There is no need for datasets to be comparable

b. The questions being asked in the research are not comparable

c. Changing current methods would weaken long-term datasets

d. Cannot cause a shift in “legacy” programs

e. Other, Please explain…

15. What is your role in the research project?

1. Principal Investigator
2. Postdoctoral researcher
3. Graduate researcher
4. Professional researcher

e) Other. Please specify…

16. What is the highest degree that you have earned and what year did you earn it?

17. What institution did or will you get your degree from?

18. Gender:

a. Male

b. Female

1. Age:

I would welcome any further discussion on this topic. Please feel free to call or email me at xxx-xxx-xxxx or email@xxx.edu
