# Supplementary material for: Reef Fish Survey Techniques: Assessing the Potential for Standardizing Methodologies
Source: PLoS One. 2016 Apr 25;11(4):e0153066. doi: 10.1371/journal.pone.0153066 (PMC4844186; doi:10.1371/journal.pone.0153066)
Supplement: S2 File — (DOCX) [file pone.0153066.s002.docx]

**S2 File**

List of Questions Included in the Survey.

1. What is the general research question that reef fish surveys help to answer in your research?
2. Where is this research involving fish surveys currently being conducted?
3. What is the purpose of the data that you are collecting?
4. What survey method is being used in your research?
5. How did you choose the methods for this project?
6. How were these methods developed?
7. If the methods were adopted or modified from another institution, what institution or organization were the methods adopted from?
8. How often are your fish surveys conducted?
9. How long have you been conducting your surveys?
10. How long do you plan to continue this research project?
11. Do you think it is valuable to have a single method across regions?
12. To what extent are you willing to modify your current methodology to produce a standardized reef fish surveying method?
13. What factors affect your willingness to modify your methods to a standardized methodology?
14. Why do you believe there has not been one standardized fish survey method adopted across all regions?
15. What is your role in the project?
16. What is your highest level of education?
17. Where did you earn your degree?
18. What is your age?
19. What is your gender?
