# Supplementary material for: Reef Fish Survey Techniques: Assessing the Potential for Standardizing Methodologies
Source: PLoS One. 2016 Apr 25;11(4):e0153066. doi: 10.1371/journal.pone.0153066 (PMC4844186; doi:10.1371/journal.pone.0153066)
Supplement: S3 File — (DOCX) [file pone.0153066.s003.docx]

**S3 File**

S3. Description of Top Three Survey Methodologies

**Belt Transect Method**

The belt transect method, also referred to as the strip transect method, is conducted as one or more observers swim along a transect line and record the fishes observed in a predetermined swath [13]. All fishes that are observed within the swath (usually between 2-5m) are tallied to the lowest recognizable taxon and size (if recorded) is estimated to nearest size class (often 1- or 5-cm bins). Transects typically run parallel to shore and follow a consistent depth within one habitat type. The speed at which the dive team moves along the transect is moderate and consistent depending on the target species that are being counted. If the dive team swims too fast, benthic dwelling or slow moving cryptic species may be overlooked resulting in an underestimate of the population. Alternatively, if the dive team swims too slowly large-bodied or highly mobile species that may enter the diver’s field of view along the transect may be overestimated [24].

**Stationary Point Count Method**

The stationary point count method is conducted as a diver records fish within a predefined cylindrical area [21]. The method generally involves two divers working as a team with each diver conducting an independent survey. The divers usually work in pairs due to diving safety standards and concerns. At the start of the count, each diver remains stationary at the center of their respective cylinder while tallying all fishes from the bottom to the top of the water column for a set amount of time. The radius of the cylinder is kept constant for the assessment, usually between 5 and 10 m, depending on visibility and targeted fish species. To account for potential bias based on differential movement patterns, larger, more mobile fishes are counted first then the more sedentary species are counted [28].

**Timed Swim Method**

The method of timed visual assessment uses time as the constant, similar to the Stationary Point Count (SPC) but without explicit constraint on the area surveyed. In some cases, the area covered can be estimated during timed swim methods through incorporation of spatial positioning equipment (e.g., towed GPS). These timed assessments most commonly focus on larger species of fishes (>20 cm) that are targeted by fishers [68], but also include rapid assessments of species diversity, for example roving diver surveys [69]. Timed visual assessments are typically used to cover a large amount of reef area with a limited amount of effort. To cover large areas of reef the divers usually swim at a rapid pace or are supported by boat-towed boards or underwater propulsion devices to maximize reef coverage [71,82].
